# Tetherin restricts direct cell-cell viral transfer and transmission of HIV-1

**DOI:** 10.1186/1758-2652-13-S3-O8

**Published:** 2010-11-04

**Authors:** BD Kuhl, RD Sloan, DA Donahue, T Bar-Magen, C Liang, MA Wainberg

**Affiliations:** 1 McGill University AIDS Center, Lady Davis Institute, Jewish General Hospital, Montréal, Canada; 2 Department of Experimental Medicine, McGill University, Montréal, Canada; 3 Department of Microbiology and Immunology, McGill University, Montréal, Canada

## Background

Tetherin (BST-2/CD317/HM1.24) is an interferon-inducible factor of the innate immune system, recently shown to exert antiviral activity against HIV-1 and other enveloped viruses by tethering nascent viral particles to the cell surface, thereby inhibiting viral release. In HIV-1 infection, the viral protein U (Vpu) counteracts this antiviral action by down-modulating tetherin from the cell surface. Viral transmission between T cells can occur via cell-free transmission or the more efficient direct cell-cell route through virological synapses. Virological synapses are associated with lipid raft-rich microdomains in the membrane. Tetherin is known to localize to these microdomains and is capable of modulating actin architecture, which is crucial for viral entry, assembly and budding.

## Methods

We established a flow cytometry-based co-culture assay to distinguish viral transfer from viral transmission and investigated the impact of tetherin on cell-cell spread of HIV-1. Sup-T1 cells inducible for tetherin expression were used to examine the impact of effector and target cell tetherin expression on virus transfer and transmission.

## Results

We show that tetherin, in addition to inhibiting viral release, also inhibits direct cell-cell virus transfer and transmission. Viral Vpu promotes viral transmission from tetherin-expressing cells by down-modulating tetherin from the effector cell surface, outweighing its possible cost of fitness in cell-cell transmission. Further, we have shown that tetherin on the target cell promotes viral transfer and transmission in a manner that favours Vpu-deficient virus.

## Conclusions

The results suggest a role for tetherin in cell-cell contacts, specifically at virological synapses. Our data further contribute to the body of knowledge that the targeting of Vpu could be a valuable strategy aimed at blocking HIV replication.

